# Bacteremia Caused by Extended-Spectrum Beta-Lactamase–Producing Enterobacteriaceae in Vientiane, Lao PDR: A 5-Year Study

**DOI:** 10.4269/ajtmh.19-0304

**Published:** 2020-03-09

**Authors:** Ko Chang, Sayaphet Rattanavong, Mayfong Mayxay, Valy Keoluangkhot, Viengmon Davong, Manivanh Vongsouvath, Manophab Luangraj, Andrew J. H. Simpson, Paul N. Newton, David A. B. Dance

**Affiliations:** 1 Adult Infectious Diseases Ward, Mahosot Hospital, Vientiane, Laos;; 2 Lao-Oxford-Mahosot Hospital-Wellcome Trust Research Unit (LOMWRU), Microbiology Laboratory, Mahosot Hospital, Vientiane, Laos;; 3 Centre for Tropical Medicine and Global Health, University of Oxford, Oxford, United Kingdom;; 4 Institute of Research and Education Development (IRED), University of Health Sciences, Vientiane, Laos;; 5 Faculty of Infectious and Tropical Diseases, London School of Hygiene and Tropical Medicine, London, United Kingdom

## Abstract

Although there has been an increasing incidence of bacteremia caused by extended-spectrum beta-lactamase (ESBL)–producing Enterobacteriaceae (ESBL-E) across South East Asia, there are sparse data from the Lao PDR, where laboratory capacity for antimicrobial resistance surveillance is limited. We, therefore, retrospectively reviewed bacteremia caused by ESBL-producing *Escherichia coli* and *Klebsiella pneumoniae* between 2010 and 2014 at Mahosot Hospital, Vientiane, Lao PDR. Clinical and laboratory data relating to all episodes of ESBL-E bacteremia were reviewed over the 5-year period and compared with non–ESBL-E bacteremia. Blood cultures positive for *E. coli* or *K. pneumoniae* were identified retrospectively from laboratory records. Clinical and laboratory data were extracted from research databases and case notes and analyzed using STATA. Between 2010 and 2014, we identified 360 patients with *E. coli* (*n* = 249) or *K. pneumoniae* (*n* = 111) bacteremia, representing 34.8% of all patients with clinically significant bacteremia*.* Seventy-two (20%) isolates produced ESBL; *E. coli* accounted for 15.3% (55/360) and *K. pneumoniae* for 4.7% (17/360), respectively. The incidence of ESBL-producing *E. coli* bacteremia rose during the study period. By multiple logistic analysis, reported antibiotic use in the previous week was significantly associated with ESBL positivity (*P* < 0.001, odds ratio 3.89). Although multiresistant, most ESBL-producing *E. coli* and *K. pneumoniae* remained susceptible to meropenem (65/65; 100%) and amikacin (64/65; 98.5%). We demonstrated an alarming increase in the incidence of ESBL-E as a cause of bacteremia in Vientiane during the study period. This has implications for empiric therapy of sepsis in Laos, and ongoing surveillance is essential.

## INTRODUCTION

Bloodstream infection is a major cause of morbidity and mortality, comparable with major traumatic injury, myocardial infarction, and stroke with an annual incidence of 140–160 per 100,000 population.^1,2^ Antimicrobial resistance (AMR) leads to increased mortality, length of hospital stay, and hospital costs associated with bloodstream infections, including both community-onset and hospital-acquired bacteremia.^3,4^ Gram-negative bacteria belonging to the family Enterobacteriaceae are associated with infections ranging from minor urinary tract infections to life-threatening bacteremia. Enterobacteriaceae, especially *Escherichia coli* and *Klebsiella pneumoniae*, are becoming a major threat to public health because of their ability to acquire resistance to most current antibiotics, especially third-generation cephalosporins, such as ceftriaxone, through the production of extended-spectrum beta-lactamases (ESBLs).^5^ The WHO has listed ESBL-producing Enterobacteriaceae (ESBL-E) as critical priority pathogens for research and development of new antibiotics.^6^ Extended-spectrum beta-lactamase–producing Enterobacteriaceae have emerged as important pathogens in many countries in the Asia–Pacific region, such as India and China where ESBL-producing isolates account for 77% and 48% of Enterobacteriaceae from intra-abdominal infections, respectively.^7^ Lao PDR is a land-locked country, surrounded by countries with high incidence rates of ESBL-E infection,^8,9^ although it has few microbiology laboratories and data on AMR are, therefore, scarce. Phetsouvanh et al.^10^ demonstrated that Gram-negative bacteria accounted for 77% of community-acquired bacteremia in Lao PDR between 2000 and 2004, with *E. coli* and *K. pneumoniae* accounting for 12% and 4%, respectively. The first ESBL-producing *E. coli* was identified in Mahosot Hospital, Vientiane, in 2004, and between 2004 and 2009, 9% of *E. coli* isolated from blood were ESBL producers.^11^ A study of preschool children in and around Vientiane in 2011 found that 23% were colonized with ESBL-E, mainly *E. coli* and *K. pneumoniae*, and colonization was strongly associated with prior antibiotic use*.*^12^ Cephalosporins, particularly ceftriaxone, are widely used for empirical treatment of febrile patients in Laos, but the lack of access to diagnostic microbiology and AMR surveillance data mean that clinicians are largely unaware of the extent of the AMR problem, leading to delays in initiating appropriate treatment. Therefore, we retrospectively reviewed the proportion, clinical features, risk factors, and antibiotic susceptibility profiles of *E. coli* and *K. pneumoniae* causing bacteremia at Mahosot Hospital, Vientiane, Laos, between 2010 and 2014.

## MATERIALS AND METHODS

Mahosot Hospital in Vientiane, Laos, is a central, tertiary hospital with 450 beds, which serves a population of approximately 800,000 people in the immediate catchment, but also acts as a referral center for other peripheral and provincial hospitals. It receives approximately 19,000 admissions per year (Mahosot Summary Report 2010–2014, unpublished data). The Mahosot Hospital Microbiology Laboratory provides a bacterial culture service for Mahosot Hospital and several other hospitals in Laos, supported by the Lao-Oxford-Mahosot Hospital-Wellcome Trust Research Unit. It receives approximately 6,500 blood culture sets annually, of which 4,600 come from inpatients at Mahosot Hospital. As part of an ongoing study of the causes of fever at Mahosot Hospital since 2000 (the “UI-study”), a set of blood cultures (two aerobic bottles [Pharmaceutical Factory Number 2, Vientiane, Laos]) is sent by the responsible clinician from all patients admitted with suspected community-acquired bacteremia, subject to written informed consent. Clinical and laboratory information at the time of study admission is recorded on standard forms and databases. Blood cultures were processed manually as described,^10^ with significant isolates identified phenotypically (by API 20E [bioMérieux, Basingstoke, United Kingdom] in the case of Enterobacteriaceae). Significant isolates were tested for antimicrobial susceptibility by disk diffusion according to the current methods of the Clinical and Laboratory Standards Institute. Any *E. coli* or *Klebsiella* spp. resistant to cefpodoxime, ceftriaxone or cefotaxime, or ceftazidime was tested for ESBL activity by disk diffusion against ceftazidime and cefotaxime with and without clavulanic acid: an increase in zone diameter ≥ 5 mm for either agent in the presence of clavulanic acid was considered as positive for ESBL.^13^ Molecular detection methods were not used in this study.

### Data collection and analysis.

We retrospectively reviewed laboratory records to identify all Mahosot Hospital patients who had blood cultures positive for *E. coli and K. pneumoniae* between 2010 and 2014. Clinical and microbiological data (including reported antibiotic use in the previous week) were exported from the UI-study database into Microsoft Excel (Microsoft Corporation, Redmond, WA). Missing and inconsistent data were checked from patient charts (where available) and primary laboratory sources (e.g., laboratory work books). Where the results could not be reconciled, the organisms were re-cultured from the stock frozen at −80°C and retested.

Data analysis was performed using STATA v 14.2 (Stata Corporation, College Station, TX). Data were summarized using descriptive statistics such as number, percentage, median, mean, and 95% CI. Categorical variables were compared using the chi-square test or Fisher’s exact test. We included the statistically significant (*P* < 0.05) and relevant variables from the univariable analysis in a multiple logistic regression model using a backward stepwise approach to identify factors associated with ESBL positivity and mortality associated with ESBL positivity.

## RESULTS

**Figure 1.**
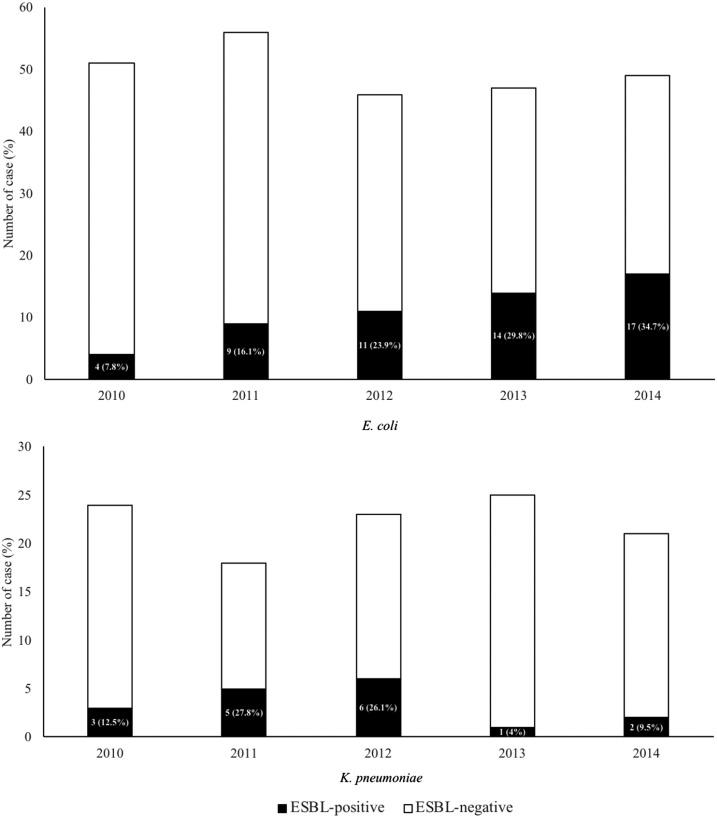
Extended-spectrum beta-lactamase (ESBL) production in *Escherichia coli* and *Klebsiella pneumoniae* blood culture isolates between 2010 and 2014 at Mahosot Hospital, Vientiane, Lao PDR.

During the period 2010–2014, the laboratory received 18,319 blood culture sets from 15,665 patients, of whom 6.6% (1,032/15,665) grew clinically significant organisms (i.e., excluding duplicates). *Escherichia coli* and *K. pneumoniae* together accounted for 34.8% (360/1,032) of all clinically significant isolates, of which 90% were classed as community-acquired bacteremia, that is, taken within 48 hours of hospital admission. Of these, 72 (20%) were ESBL positive and 288 (80%) were ESBL negative. In the ESBL-producing group, *E. coli* and *K. pneumoniae* accounted for 15.3% (55/360) and 4.7% (17/360), respectively. The proportion of ESBL-producing *E. coli* was greater than ESBL-producing *K. pneumoniae* (15.3% versus 4.7%, *P* < 0.001) throughout the study period. The proportion of *E. coli*-producing ESBL increased more than 4-fold from four (7.8%) cases in 2010 to 17 (34.7%) cases in 2014 (Figure 1).

Basic clinical and epidemiological features of the patients are shown in Table 1. Of the 360 patients, 143 (40%) were male and the overall median interquartile range (IQR) age was 57 (42–70) years. Patients with ESBL-positive infections were significantly younger than those with ESBL-negative infections (*P* = 0.03). Most of the patients presented with acute illness (median [IQR] days of illness ∼ 3 [2–7]) and there was no difference between the ESBL-positive and ESBL-negative groups in terms of clinical presentation.

**Table 1:** Characteristics and symptoms of patients with ESBL-negative and ESBL-positive *Escherichia coli* and *Klebsiella pneumoniae* bacteremia at the time of study admission (data shown as number (%) unless indicated)

	Total (*n* = 360)	ESBL negative (*n* = 288)	ESBL positive (*n* = 72)	*P*-value
Demographic data				
Age (years), median (IQR)	57 (42–70)	58 (43–70)	53 (32–65)	0.03
≤ 15 years old, *n*/*N* (%)	27/360 (8)	18/288 (6)	9/72 (13)	0.09
Male, *n*/*N* (%)	143/360 (40)	108/288 (38)	35/72 (49)	0.09
Symptoms				
Days ill, median (IQR)	3 (2–7)	3 (2–7)	3.5 (2–7)	0.70
Fever, *n*/*N* (%)	345/356 (97)	277/286 (97)	68/70 (97)	1.00
Rigors, *n*/*N* (%)	230/354 (65)	190/285 (67)	40/69 (58)	0.17
Headache, *n*/*N* (%)	197/355 (55)	161/286 (56)	36/69 (52)	0.50
Arthralgia, *n*/*N* (%)	118/355 (33)	95/286 (33)	23/69 (33)	0.90
Back pain, *n*/*N* (%)	123/355 (35)	99/286 (35)	24/69 (35)	0.90
Myalgia, *n*/*N* (%)	184/355 (52)	149/286 (52)	35/69 (51)	0.80
Retro-orbital pain, *n*/*N* (%)	16/340 (5)	15/275 (6)	1/65 (2)	0.32
Jaundice, *n*/*N* (%)	64/351 (18)	51/284 (18)	13/67 (19)	0.70
Nausea, *n*/*N* (%)	121/352 (34)	101/284 (36)	20/68 (29)	0.30
Vomiting, *n*/*N* (%)	99/352 (28)	82/284 (28)	17/68 (25)	0.50
Dysuria, *n*/*N* (%)	62/352 (17)	49/284 (17)	13/68 (19)	0.71
Diarrhea, *n*/*N* (%)	67/351 (19)	59/283 (21)	8/68 (12)	0.08
Constipation, *n*/*N* (%)	25/349 (7)	21/282 (7)	4/67 (6)	0.79
Abdominal pain, *n*/*N* (%)	100/346 (29)	84/280 (30)	16/66 (24)	0.30
Sore throat, *n*/*N* (%)	14/346 (4)	11/280 (4)	3/66 (5)	0.78
Drowsiness, *n*/*N* (%)	56/346 (16)	43/280 (15)	13/66 (20)	0.38
Risk factors, *n*/*N* (%)				
Diabetes, *n*/*N* (%)	88/335 (26)	76/272 (28)	12/63 (19)	0.31
Excessive alcohol use, *n*/*N* (%)	34/317 (11)	27/257 (11)	7/60 (12)	0.79
Smoking, *n*/*N* (%)	37/311 (12)	28/253 (11)	9/58 (16)	0.34
Chronic renal failure, *n*/*N* (%)	18/341 (5)	11/278 (4)	7/64 (11)	0.05
Renal calculi, *n*/*N* (%)	20/336 (6)	12/275 (4)	8/61 (13)	0.01
TB, *n*/*N* (%)	6/236 (3)	5/195 (3)	1/42 (2)	0.92
HIV, *n*/*N* (%)	5/294 (2)	5/243 (2)	0/52 (0)	0.72
Steroid use, *n*/*N* (%)	14/303 (5)	11/252 (4)	3/52 (6)	0.80
Reported antibiotic use in the previous week, *n*/*N* (%)	61/235 (26)	38/192 (20)	23/44 (52)	< 0.001
Outcome				
Discharged alive, *n*/*N* (%)	144/203 (71)	122/164 (74)	22/39 (56)	0.04
Died in hospital, *n*/*N* (%)	25/203 (12)	16/164 (10)	9/39 (23)	
Discharged moribund, *n*/*N* (%)	34/203 (17)	26/164 (16)	8/39 (20)	

ESBL = extended-spectrum beta-lactamase.

Details of risk factors and outcomes are shown in Table 1. By univariable analysis, the presence of renal calculi (odds ratio [OR] 3.38, 95% CI: 1.20–9.48), chronic renal failure (OR 3.06, 95% CI: 1.14–8.24) and reported antibiotic use in the previous week (OR 4.40, 95% CI: 2.21–8.79) were significantly associated with ESBL positivity (*P* < 0.05 for all). However, by multiple logistic regression, only reported antibiotic use in the previous week was independently significantly associated with ESBL positivity (*P* < 0.001, OR 3.89, 95% CI: 1.85–8.18).

Unfortunately, only 203 (56%) of 360 patients had outcome data available; 144/203 (71%) were discharged alive, but the proportion of patients who died or were discharged moribund (a common outcome in Laos for cultural reasons) was significantly higher in the ESBL-positive group than in the ESBL-negative patients. However, patient outcomes were not independently associated with ESBL positivity by multiple logistic regression (*P* = 0.21). In the univariate analysis, patients infected with ESBL-producing *K. pneumoniae* were significantly younger and less frequently reported a history of fever compared with those infected with ESBL-producing *E. coli.* There were no other significant differences between two pathogens in term of clinical presentations, co-morbidities, and outcomes (Table 2).

**Table 2:** Comparison of characteristics and symptoms of patients with ESBL-positive *Escherichia coli* and *Klebsiella pneumoniae* bacteremia (data shown as number (%) unless indicated)

	Total (*n* = 72)	ESBL—*E. coli* (*n* = 55)	ESBL—*K. pneumoniae* (*n* = 17)	*P*-value
Demographic data				
Age (years), median (IQR)	53 (32–65)	56 (45–65)	24 (0.41–54)	0.0097
≤ 15 year old, *n*/*N* (%)	9/72 (12)	2/55 (3.6)	7/17 (41)	< 0.001
Male, *n*/*N* (%)	35/72 (49)	26/55 (47)	9/17 (53)	0.68
Symptoms				
Days ill, median (IQR)	3.5 (2–7)	3 (2–7)	4 (2–7)	0.77
Fever, *n*/*N* (%)	68/70 (97)	55/55 (100)	13/15 (86)	0.04
Rigors, *n*/*N* (%)	40/69 (58)	34/54 (63)	6/15 (40)	0.11
Headache, *n*/*N* (%)	36/69 (52)	31/54 (57)	5/15 (33)	0.09
Arthralgia, *n*/*N* (%)	23/69 (33)	19/54 (35)	4/15 (27)	0.75
Back pain, *n*/*N* (%)	24/69 (35)	18/54 (33)	6/15 (40)	0.63
Myalgia, *n*/*N* (%)	35/69 (51)	30/54 (55)	5/15 (33)	0.12
Retro-orbital pain, *n*/*N* (%)	1/65 (2)	1/50 (2)	0/15 (0)	1.00
Jaundice, *n*/*N* (%)	13/67 (19)	12/52 (23)	1/15 (7)	0.26
Nausea, *n*/*N* (%)	20/68 (29)	17/53 (32)	3/15 (20)	0.52
Vomiting, *n*/*N* (%)	17/68 (25)	14/53 (26)	3/15 (20)	0.74
Dysuria, *n*/*N* (%)	13/68 (19)	11/53 (21)	2/15 (13)	0.71
Diarrhea, *n*/*N* (%)	8/68 (12)	6/53 (11)	2/15 (13)	1.00
Constipation, *n*/*N* (%)	4/67 (6)	4/53 (7)	0/14 (0)	0.57
Abdominal pain, *n*/*N* (%)	16/66 (24)	12/51 (23)	4/15 (27)	1.00
Sore throat, *n*/*N* (%)	3/66 (5)	2/51 (4)	1/15 (7)	0.54
Drowsiness, *n*/*N* (%)	13/66 (20)	10/51 (19)	3/15 (20)	1.00
Risk factor				
Diabetes, *n*/*N* (%)	12/63 (19)	9/51 (17)	3/12 (25)	0.25
Excess alcohol, *n*/*N* (%)	7/60 (12)	6/48 (12)	1/12 (8)	1.00
Smokes, *n*/*N* (%)	9/58 (15)	8/46 (17)	1/12 (8)	0.66
Chronic renal failure, *n*/*N* (%)	7/64 (11)	6/50 (12)	1/14 (7)	0.91
Renal calculi, *n*/*N* (%)	8/61 (13)	7/47 (15)	1/14 (7)	0.63
Steroid use, *n*/*N* (%)	3/52 (6)	2/41 (5)	1/11 (9)	0.75
Reported antibiotic use in the previous week, *n*/*N* (%)	23/44 (52)	18/35 (51)	5/9 (56)	1.00
Outcome				
Discharged alive, *n*/*N* (%)	22/39 (56)	16/28 (57)	6/11 (55)	0.79
Died in hospital, *n*/*N* (%)	9/39 (23)	7/28 (25)	2/11 (18)	
Discharged moribund, *n*/*N* (%)	8/39 (20)	5/28 (18)	3/11 (27)	

ESBL = extended-spectrum beta-lactamase.

All ESBL-producing *E. coli* and *K. pneumoniae* in this study were susceptible to imipenem and/or meropenem (Table 3). The proportion susceptible to gentamicin declined sharply in 2011 but was relatively stable from 2012 to 2014, whereas all isolates were sensitive to amikacin apart from a single isolate in 2014 from a patient with biliary sepsis. For other groups of antibiotics, susceptibility rates were relatively consistent, with no obvious trend during the study period: chloramphenicol was active against 47–68% of isolates, followed by ofloxacin and ciprofloxacin (33–57% susceptible). Fewer than half of the isolates appeared susceptible in vitro to co-amoxiclav and fewer than 30% to ceftazidime and co-trimoxazole.

**Table 3:** Antimicrobial susceptibility of extended-spectrum beta-lactamase–producing *Escherichia coli* and *Klebsiella pneumoniae* isolated from blood cultures between 2010 and 2014 at Mahosot Hospital, Vientiane, Lao PDR

Year	2010	2011	2012	2013	2014	*P*-value
Antimicrobial	*n*/*N* (%)	*n*/*N* (%)	*n*/*N* (%)	*n*/*N* (%)	*n*/*N* (%)	
Ampicillin	0/7 (0)	0/14 (0)	0/17 (0)	0/15 (0)	0/19 (0)	NA
Co-amoxiclav	2/7 (29)	3/14 (21)	8/17 (47)	7/15 (47)	4/19 (21)	0.55
Cefalotin	0/7 (0)	0/14 (0)	0/17 (0)	0/15 (0)	0/19 (0)	NA
Ceftazidime	1/5 (20)	3/12 (25)	4/14 (28)	2/15 (13)	4/19 (21)	0.96
Ceftriaxone	0/7 (0)	0/14 (0)	0/17 (0)	0/15 (0)	0/19 (0)	NA
Ciprofloxacin	1/1 (100)	ND	ND	6/13 (46)	5/15 (33)	0.81
Chloramphenicol	4/7 (57)	7/14 (50)	8/17 (47)	9/15 (60)	13/19 (68)	0.51
Doxycycline	ND	ND	ND	1/4 (25)	1/3 (33)	1.00
Gentamicin	5/7 (71)	3/14 (21)	8/17 (47)	8/15 (53)	10/19 (52)	0.15
Meropenem	6/6 (100)	12/12 (100)	15/15 (100)	15/15 (100)	17/17 (100)	NA
Imipenem	7/7 (100)	13/13 (100)	13/13 (100)	15/15 (100)	15/15 (100)	NA
Ofloxacin	4/7 (57)	5/14 (36)	9/17 (53)	8/15 (53)	6/19 (31)	0.53
Co-trimoxazole	0/7 (0)	3/14 (21)	4/17 (23)	4/15 (27)	3/19 (16)	0.70
Tetracycline	ND	1/1 (100)	ND	3/11 (27)	2/11 (18)	0.37
Amikacin	6/6 (100)	12/12 (100)	15/15 (100)	15/15 (100)	16/17 (94)	1.00

*n* = number of isolates susceptible to tested antimicrobial; *N* = total number of isolates tested; ND = not done; NA = not applicable. Figures in parentheses are percentages. Variations in denominators reflect the fact that not all isolates were tested against each agent.

## DISCUSSION

Since 2000, bloodstream isolates of *E. coli* and *K. pneumoniae* have been routinely screened for ESBL production at Mahosot Hospital. Results are reported to clinicians in real time. Since the first ESBL-producing *E. coli* identified in 2004, there has been a steady increase in the proportion of bacteremia caused by ESBL-E in Vientiane. We previously reported that 9% of *E. coli* causing bloodstream infection between 2004 and 2009 were ESBL producers.^11^ This retrospective study confirms that the overall proportion of Enterobacteriaceae causing bacteremia that produce ESBL further increased 3-fold from 2010 to 2014.

All ESBL-E should not be considered as a homogeneous group.^14^ The reasons for the differences are unclear, but imply distinct epidemiological differences between the two species. Extended-spectrum beta-lactamase-producing *E. coli* was significantly more common than ESBL-producing *K. pneumoniae* as a cause of bacteremia in our population (15.28% versus 4.72%, *P* < 0.001). Interestingly, our study demonstrated different chronological trends for ESBL-producing *E. coli* bacteremia, which increased in proportion nearly 5-fold during the study period, and ESBL-producing *K. pneumoniae* bacteremia, which initially doubled (from 12.5% in 2010 to 26.1% in 2012) but then declined to 4% and 9.5% in 2013 and 2014, respectively. Similar observations have been made in other studies.^15^ We also observed that patients with ESBL-producing *K. pneumoniae* bacteremia were younger than those with ESBL-producing *E. coli* bacteremia, which has also been described by others.^16^

Overall, this increase in the frequency of bacteremia caused by ESBL-E has significant implications for the empirical management of community-acquired sepsis, which is usually treated with ceftriaxone in Laos. Moreover, most of these isolates were multiresistant, leading to severe problems in selecting appropriate agents to replace ceftriaxone. However, the picture is not entirely bleak, as the proportion of ESBL-E as causes of bacteremia is still lower in Laos than in some neighboring countries, which have been high for many years.^7^ The proportion of *E. coli* and *K. pneumoniae* causing community-acquired bacteremia that are ESBL positive varies between countries across South East Asia, ranging from 11.8% to 50% for *E. coli* and 11.4% to 43.8% for *K. pneumoniae,* respectively.^17–20^ For example, between 2004 and 2010, a retrospective, multicenter surveillance study in all provincial hospitals in northeast Thailand, which lies to the south and west of Laos across the Mekong river, found that ESBL was produced by 11.8% of *E. coli* and 11.4% of *K. pneumoniae* causing bacteremia, with an increasing year-on-year trend.^17^ In Cambodia between July 2007 and December 2010, 47.7% of *E. coli* and 43.8% of *K. pneumoniae* causing bacteremia produced ESBL.^18^ A retrospective study conducted at the National Hospital for Tropical Diseases in Hanoi, Vietnam, between January 2011 and December 2013 demonstrated that the proportions of *E. coli* and *K. pneumoniae* causing bacteremia that produced ESBL were 45% and 12.3%, respectively.^19^ A more recent study conducted at three hospitals in Yangon, Myanmar, from July to December 2014, found that 50% of *E. coli* and 43% of *K. pneumoniae* causing bacteremia produced ESBL.^20^ In a prospective multicenter study conducted in 28 tertiary hospitals across China from September 2013 to November 2014, the corresponding proportions were 55.5% and 16.7%, respectively.^21^ The fact that Laos is still lagging behind these countries means that there may be a chance to intervene before the problem reaches the size of that in its neighbors. This should inform rapid policy decisions and implementation to prevent the situation worsening.

Our retrospective study found a number of factors that were associated with ESBL production in patients with *E. coli* and *K. pneumoniae* bacteremia, including renal calculi, chronic renal failure, and reported antibiotic use in the previous week. However, only reported antibiotic use in the previous week remained as a significant risk factor by multiple logistic regression. The recorded antibiotic use data did not, however, include the details of antibiotic class, length of, or reasons for prescription. Previous studies have also shown that healthcare-associated infection, obstructive urinary tract disease, chronic kidney disease, cerebrovascular disease, heart failure, previous major surgery, malignancies, and previous use of antibiotics were associated with bloodstream infection caused by ESBL-producing *E. coli* and *K. pneumoniae*.^21–26^ Furthermore, prior use of several different classes of antimicrobials, including extended-spectrum cephalosporins, fluoroquinolones, aminoglycosides, co-trimoxazole, and carbapenems, has repeatedly been associated with ESBL-producing *E. coli* and *K. pneumoniae* bacteremia,^23,24,27–32^ and a study from Spain showed that receipt of more than two different antibiotic classes in the preceding 90 days was the only predictor of ESBL production in patients with *E. coli* or *K. pneumoniae* bacteremia (OR 2.29, 95% CI: 1.35–3.88).^33^ Whether the link observed in our study was causal or was simply a reflection of the fact that blood cultures are more likely to be positive in patients on antibiotics who are infected with multiresistant organisms than with susceptible organisms is impossible to say. However, inappropriate antibiotic prescribing is common in Laos and there is a clear need to educate local clinicians about diagnostic stewardship and the prudent use of antibiotics.^34^

Several studies have demonstrated that the mortality rate associated with ESBL-producing *E. coli* and *K. pneumoniae* bacteremia is significantly (30–50%) higher than that of non–ESBL-producing organisms,^35–38^ as it was in our study and associated with longer hospital stays and costs.^39^ The selection of appropriate antimicrobial therapy is an essential intervention to improve patient outcomes.^40^ This is particularly important during empirical treatment, although susceptibility results are still pending or in patients who are critically ill and are not responding to initial treatment with agents such as third-generation cephalosporins. This study has shown that local ESBL-producing *E. coli* and *K. pneumoniae* causing bacteremia in Vientiane have a high rate of co-resistance to other groups of antibiotics but remain susceptible to meropenem and mostly to amikacin. This is consistent with previous studies from other centers.^41–43^ The suggested empirical treatment for patients with suspected sepsis in Mahosot Hospital is currently ceftriaxone,^44^ but this study raises the question as to whether this should be changed. However, carbapenems are expensive and not readily available in Laos and amikacin, although often used when indicated, is potentially toxic, especially as therapeutic drug monitoring is not locally available. There is also a risk of the emergence of resistance if these agents were more widely used.^45–47^ Carbapenemase screening is already in place in Mahosot Hospital. We are currently investigating the use of rapid methods to detect ESBL production in patients with positive blood cultures growing Gram-negative bacilli in the hope that more targeted use of these valuable agents may be possible.

Our study has several limitations. Because of its retrospective nature, some hospital charts could not be retrieved, and so it was not possible always to capture data about risk factors, comorbidities, and clinical outcomes related to ESBL-E bacteremia. Second, this study was carried out in a single center and might not be applicable to other settings across Laos. Third, the estimated number of presumed hospital-acquired bacteremias is small and possibly exaggerated (maximum 10% overall) and we have, therefore, not attempted to analyze the data by whether episodes were hospital or community acquired. Furthermore, blood cultures are not always collected on admission. Reported antibiotic use data are also limited in scope, particularly timings and class. Last, the data relate to 2010–2014 and because the epidemiology of AMR is likely to be changing constantly, expanded surveillance is essential.

## CONCLUSION

Our study has demonstrated an alarming increase in the incidence of ESBL-producing *E. coli* and, to a lesser extent *K. pneumoniae*, bacteremia in Laos. Because most isolates were multiresistant, the effectiveness of antibiotics that are commonly used in the local setting is compromised. This emphasizes the need for appropriate local antibiotic guidelines based on accurate knowledge of local resistance patterns to improve patient outcomes and the importance of efforts to promote antimicrobial stewardship in Laos before the levels of AMR reach those of neighboring countries.

In addition, more detailed and ongoing AMR surveillance and research in the Lao PDR are also required, to build on this limited study.
